# The Molecular Basis of High-Altitude Adaptation in Deer Mice

**DOI:** 10.1371/journal.pgen.0030045

**Published:** 2007-03-30

**Authors:** Jay F Storz, Stephen J Sabatino, Federico G Hoffmann, Eben J Gering, Hideaki Moriyama, Nuno Ferrand, Bruno Monteiro, Michael W Nachman

**Affiliations:** 1 School of Biological Sciences, University of Nebraska, Lincoln, Nebraska, United States of America; 2 Department of Chemistry, University of Nebraska, Lincoln, Nebraska, United States of America; 3 Centro de Investigação em Biodiversidade e Recursos Genéticos, Campus Agrário de Vairão, Universidade do Porto, Vairão, Portugal; 4 Departamento de Zoologia e Anthropologia, Faculdade de Ciências do Porto, Porto, Portugal; 5 Department of Ecology and Evolutionary Biology, University of Arizona, Tucson, Arizona, United States of America; University of Chicago, United States of America

## Abstract

Elucidating genetic mechanisms of adaptation is a goal of central importance in evolutionary biology, yet few empirical studies have succeeded in documenting causal links between molecular variation and organismal fitness in natural populations. Here we report a population genetic analysis of a two-locus α-globin polymorphism that underlies physiological adaptation to high-altitude hypoxia in natural populations of deer mice, Peromyscus maniculatus. This system provides a rare opportunity to examine the molecular underpinnings of fitness-related variation in protein function that can be related to a well-defined selection pressure. We surveyed DNA sequence variation in the duplicated α-globin genes of P. maniculatus from high- and low-altitude localities (i) to identify the specific mutations that may be responsible for the divergent fine-tuning of hemoglobin function and (ii) to test whether the genes exhibit the expected signature of diversifying selection between populations that inhabit different elevational zones. Results demonstrate that functionally distinct protein alleles are maintained as a long-term balanced polymorphism and that adaptive modifications of hemoglobin function are produced by the independent or joint effects of five amino acid mutations that modulate oxygen-binding affinity.

## Author Summary

A major goal in evolutionary biology is to identify the specific genetic mechanisms that have enabled organisms to adapt to their environments. Variation in deer mouse hemoglobin represents an especially promising system for examining the molecular underpinnings of adaptation because it has been possible to establish a mechanistic link between allelic variation in protein function and fitness-related variation in physiological performance. Specifically, adaptive variation in blood biochemistry and aerobic metabolism among mice from different elevations is associated with allelic variation at two closely linked gene duplicates that encode the α-chain subunits of adult hemoglobin. In this study, we report an analysis of DNA sequence variation in the two α-globin gene duplicates of deer mice in order to identify the specific mutations that underlie adaptation to high-altitude hypoxia. The study revealed that allelic differences in hemoglobin-oxygen affinity are attributable to the independent or joint effects of substitutions in five exterior amino acid residues that line the opening of the heme pocket. Additionally, patterns of DNA sequence variation indicate that functionally distinct α-globin alleles are maintained by natural selection that favors different genotypes in different elevational zones.

## Introduction

Many long-standing questions about genetic mechanisms of adaptation remain unanswered due to the difficulty of integrating molecular data with evidence for causal effects on organismal fitness. In principle, progress could be made by identifying key proteins or key components of protein interaction networks that are known to mediate an adaptive response to some specific environmental challenge. Analysis of DNA sequence variation at the underlying genes could then guide the identification of specific nucleotide changes that are responsible for functional modifications of biochemical or physiological pathways, and could also shed light on the role of natural selection in maintaining the observed variation in protein function [1,2]. Although this approach holds much promise, very few studies have successfully documented a mechanistic link between allelic variation in protein function and fitness-related variation in whole-organism physiology [3–7].

Hemoglobin polymorphism in the deer mouse, *Peromyscus maniculatus,* represents an especially promising system for examining the molecular underpinnings of physiological adaptation to different environments. P. maniculatus has the broadest altitudinal range of any North American mammal, as the species is continuously distributed from sea-level environments to alpine environments at elevations above 4,300 m. At 4,300 m, the partial pressure of oxygen (Po_2_) is approximately 55% of the sea-level value, and the resultant hypoxia imposes severe constraints on aerobic metabolism. Experimental evidence indicates that adaptive variation in blood biochemistry among mice from different elevations is associated with a complex hemoglobin polymorphism [8–11]. Specifically, experimental crosses involving wild-derived strains of P. maniculatus revealed that variation in blood oxygen affinity is strongly associated with allelic variation at two closely linked gene duplicates that encode the α-chain subunits of adult hemoglobin [12,13]. In *P. maniculatus,* the two α-globin gene duplicates, *Hba* and *Hbc,* are each polymorphic for two main classes of electrophoretically detectable protein alleles, *Hba* ^0^, *Hba* ^1^, *Hbc* ^0^, and *Hbc* ^1^ [8,9,14,15]. These loci are closely linked, and because of strong linkage disequilibrium, nearly all α-globin haplotypes fall into two main classes: *a* ^0^ *c* ^0^ and *a* ^1^ *c* ^1^. The three nonrecombinant genotypes exhibit a highly consistent rank-order of blood oxygen affinities when tested under both high- and low-altitude conditions: mice with the *a* ^0^ *c* ^0^/*a* ^0^ *c* ^0^ genotype exhibit the highest affinity (the most left-shifted oxygen dissociation curve), mice with the *a* ^1^ *c* ^1^/*a* ^1^ *c* ^1^ genotype exhibit the lowest affinity (the most right-shifted dissociation curve), and the *a* ^0^ *c* ^0^/*a* ^1^ *c* ^1^ double heterozygotes are intermediate [12,13]. In these experiments, the wild-derived strains of mice carried different α-globin haplotypes in identical-by-descent condition, and the effects of the two genes were isolated against a randomized genetic background.

In addition to the effects on blood biochemistry, the phenotypic effects of these α-globin genes are also manifest at the level of whole-organism physiology. In the context of adaptation to high-altitude hypoxia, one especially important measure of physiological performance is Vo_2max_, which is defined as the maximal rate of oxygen consumption elicited by aerobic exercise or cold exposure. Vo_2max_ sets the upper limit on two important types of physiological performance: capacity for sustained activity (aerobic capacity) and internal heat production (thermogenic capacity). This measure of aerobic performance shows a striking pattern of variation among mice with different α-globin genotypes: Vo_2max_ is highest for *a* ^0^ *c* ^0^/*a* ^0^ *c* ^0^ mice when tested at an altitude of 3,800 m, whereas Vo_2max_ is highest for *a* ^1^ *c* ^1^/*a* ^1^ *c* ^1^ mice when tested at 340 m [12,13].

Consistent with predictions based on physiological considerations, survivorship studies of free-ranging P. maniculatus revealed that Vo_2max_ is subject to strong directional selection in high-altitude populations of this species [16]. This system therefore fulfills several key requirements for a comprehensive study of adaptive evolution at the molecular level [3], as there are clearly defined connections between genotype, phenotype, and Darwinian fitness in natural populations.

The physiological data indicate that the high-affinity *a* ^0^ *c* ^0^/*a* ^0^ *c* ^0^ genotype is associated with superior physiological performance under hypoxic conditions at high altitude but is associated with poor performance (relative to the *a* ^1^ *c* ^1^/*a* ^1^ *c* ^1^ genotype) in the oxygen-rich environment at sea level. In both altitudinal extremes, the *a* ^0^ *c* ^0^/*a* ^1^ *c* ^1^ heterozygotes are generally intermediate with respect to both blood oxygen affinity and Vo_2max_ [12,13]. This rank order of genotypic effects appears to be attributable to the fact that the possession of high-affinity hemoglobin facilitates pulmonary oxygen loading in high-altitude environments that are characterized by a low Po_2_ but hinders the release of oxygen to metabolizing tissues in low-altitude environments with relatively high Po_2_.

In summary, this system represents a unique case where fitness-related variation in whole-organism physiology can be related to a relatively simple biochemical phenotype (blood oxygen affinity) that has a well-characterized genetic basis. Thus, examination of DNA sequence variation at the underlying α-globin genes should reveal the specific molecular changes that underlie physiological adaptation to high-altitude hypoxia. For example, it may be possible to determine whether adaptive modifications of hemoglobin function involve just a few amino acid mutations of large effect or many mutations of individually small effect [3,17]. Mutations of large effect might be expected to involve heme–protein contacts, intersubunit contacts, or binding sites for heterotropic ligands that modulate hemoglobin-oxygen affinity [17–20]. By contrast, the gradual accumulation of minor mutations may produce adaptive shifts in oxygen affinity through more subtle and indirect effects on the stereochemistry of ligand binding. In addition to elucidating molecular mechanisms of physiological adaptation, the analysis of DNA sequence variation at fitness-related genes should provide insights into the mode of selection that is responsible for maintaining variation in protein function.

Here we report a population genetic analysis of the two-locus α-globin polymorphism in high- and low-altitude samples of P. maniculatus. The objectives are (i) to identify the specific mutations that may be responsible for the divergent fine-tuning of hemoglobin function and (ii) to test whether patterns of nucleotide variation at the two α-globin genes exhibit the expected signature of diversifying selection between high- and low-altitude populations.

## Results/Discussion

We sampled a total of 41 mice from three localities along an altitudinal transect that spanned the interface between the Great Plains and the Front Range of the Rocky Mountains in North America (see Materials and Methods). The transect spanned 3,727 m of vertical relief over a linear distance of 547 km, from prairie grassland in Kansas (620 m) to the summit of Mt. Evans, Colorado (4,347 m).

We cloned two paralogous copies of adult α-globin by screening a P. maniculatus genomic library (see Materials and Methods). By conducting thin-layer isoelectric focusing (IEF) analysis on hemolysates from wild-caught P. maniculatus and by matching IEF α-globin genotypes to the deduced amino acid sequences for the same individuals, we confirmed that the 5′ and 3′ α-globin genes are referable to the previously described *Hba* and *Hbc* genes, respectively [8–10,12–15]. Based on electrophoretic band densities, we also confirmed that 5′ α-chain tetramers constitute the major fraction of adult hemoglobin in deer mouse erythrocytes and that 3′ α-chain tetramers constitute the minor fraction [8,14,15,21].

### Gene Conversion and Sequence Divergence between the Two α-Globin Paralogs

Analysis of DNA sequence variation revealed that the two α-globin paralogs of P. maniculatus have experienced a history of biased gene conversion: 32.8% of the sampled 3′ alleles have been partially converted by 5′ α-globin (median tract length, 114 base pairs [bp]; range, 3 to 257 bp), and 10.8% of the sampled 5′ alleles have been partially converted by 3′ α-globin (median tract length, 29 bp; range, 13 to 225 bp).

**Figure 1:**
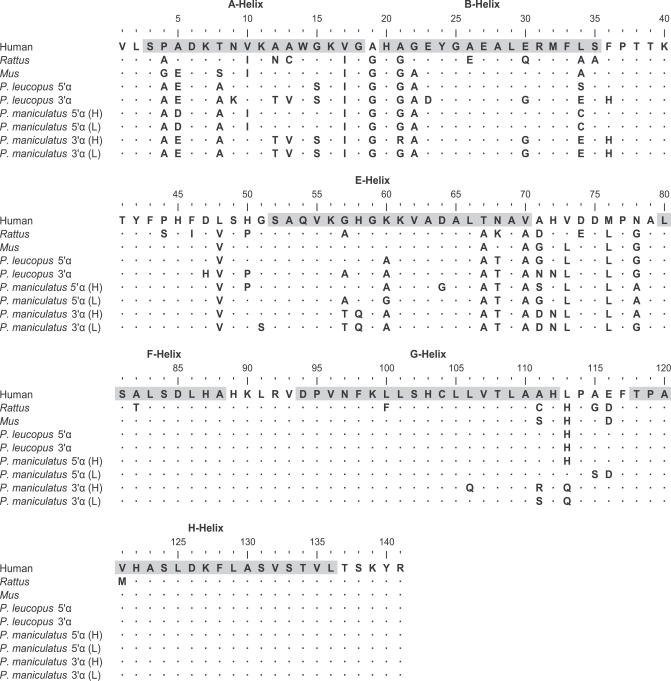
Structural Alignment of Mammalian α-Globins, including Representative High- and Low-Altitude Protein Alleles (Denoted by H and L, Respectively) from Both Paralogs of P. maniculatus

Excluding alleles with identified gene conversion tracts, the two α-globin paralogs of P. maniculatus are distinguished by eight fixed differences at nonsynonymous sites, which result in six amino acid substitutions because there are two separate instances in which a pair of nonsynonymous mutations alter the same codon: 34(B15)Cys/Ser→Glu, 36(CD1)Phe/Ser→His, 57(E6)Gly/Ala→Thr, 58(E7)His→Gln, 71(EF1)Gly/Ser→Asp, and 72(EF2)His→Asn (Figure 1). Of the six amino acid substitutions that distinguish the two α-globin paralogs, the 58(E7)His→Gln substitution in the 3′ α-globin gene is predicted to have the most important functional consequences with respect to ligand binding kinetics. In all mammals studied to date, the E7 residue of the highly conserved E-helix domain is a histidine that plays a critical role in the reversible binding of oxygen to the heme iron. Specifically, the N_ɛ_ atom of the imidazole side-chain forms a hydrogen bond with the free atom of the bound oxygen molecule [22–24]. In the 3′ α-globin gene of *P. maniculatus,* the substitution of glutamine for histidine at this position increases the N_ɛ_–O bond distance by 1.1 to 1.9 Å, thereby producing a minor shift in the three-dimensional coordinates of the heme–ligand complex (Table 1). Protein engineering studies of human hemoglobin have revealed that this 58(E7)His→Gln substitution results in an increased oxygen-binding affinity at low Po_2_ relative to wild-type (E7)His-containing hemoglobin [25]. The important physiological implication is that the erythrocytes of deer mice contain a heterogeneous mixture of (E7)His- and (E7)Gln-containing hemoglobin isoforms that have different oxygen-binding affinities. The possession of multiple adult hemoglobin isoforms with different oxygen affinities has only been documented in one other mammal, the yak, Bos grunniens [26], which inhabits high alpine environments at elevations of up to 5,400 m on the Tibetan Plateau. The possession of multiple hemoglobin isoforms has also been documented in birds that fly at extremely high altitudes [17,19,27–30]. These patterns suggest the hypothesis that the possession of multiple hemoglobin isoforms provides a mechanism for fine-tuning blood oxygen affinity in response to ambient variation in oxygen tension (in the case of animals that experience different oxygen environments on a daily or seasonal basis) or in response to variation in metabolic demands in a uniformly hypoxic environment. In the case of deer mice, an obvious prediction of this hypothesis is that some mechanism of regulatory control allows the stoichiometric ratio of 5′ and 3′ α-chain hemoglobin isoforms to be adjusted in response to metabolic demand.

**Table 1:**
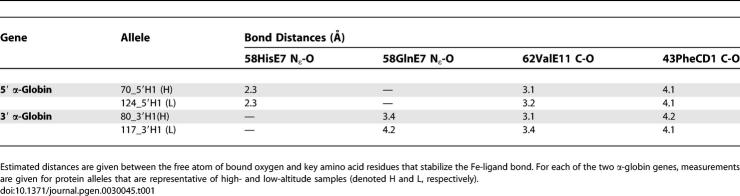
Interatomic Distances in the Heme–Ligand Complex of Deer Mouse Hemoglobin, Inferred from a Homology-Based Model

Functional divergence between the two α-globin paralogs of P. maniculatus stands in stark contrast to the pattern of concerted evolution that has been documented for the α-globin paralogs of almost all other mammals studied to date [31]. For example, the α-globin gene duplicates of humans are typically identical in sequence and therefore encode identical polypeptides [32].

### Patterns of Polymorphism and Divergence

Even after accounting for shared polymorphisms that are attributable to gene conversion between the two α-globin paralogs, both genes are characterized by extremely high levels of nucleotide diversity at both silent sites (synonymous and noncoding) and replacement sites (Table 2). In the 3′ α-globin gene, conversion tracts encompass large portions of the coding sequence and result in a 1.51-fold increase in total nucleotide diversity. In addition to the variation introduced by gene conversion between the two paralogs, both genes have also experienced high rates of intragenic recombination (*R*_M_, the inferred minimum number of recombination events = 14 for 5′ α-globin, 95% confidence interval = 6 to 16, and *R*_M_ = 8 for 3′ α-globin, 95% confidence interval = 5 to 14).

**Table 2:**
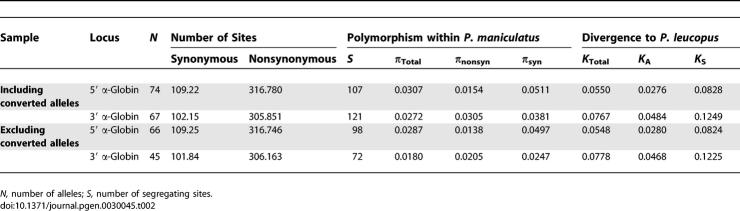
Polymorphism and Divergence in the Coding Regions of the 5′ and 3′ α-Globin Paralogs

The 3′ α-globin orthologs of P. maniculatus and a closely related congener, *Peromyscus leucopus,* are distinguished by seven amino acid substitutions: 23(B4)Asp→Gln, 47(CD12)His→Asp, 50(CD15)Pro→His, 57(E6)Gly→Thr, 58(E7)Gln→His, 71(EF1)Asn→Asp, and 113(GH1)His→Gln (Figure 1). By contrast, there are no fixed amino acid differences between the 5′ α-globin orthologs of these two species. Within *P. maniculatus,* both genes exhibit extensive amino acid replacement polymorphism: 5′ α-globin segregates 21 nonsynonymous mutations and 3′ α-globin segregates 33 nonsynonymous mutations. For neutral genes that are evolving under the joint influence of mutation and genetic drift, the level of within-species polymorphism is expected to be positively correlated with the level of between-species divergence, and this correlation should hold for both silent and replacement changes [33]. To test for a departure from this neutral expectation, we used Fisher's exact test of independence to assess whether the ratio of amino acid replacement to silent polymorphisms in each of the two α-globin genes of P. maniculatus differed from the ratio of replacement to silent fixed differences in comparison with their respective orthologs in P. leucopus. Excluding alleles with identified gene conversion tracts, the tests revealed that both genes are characterized by a significant excess of replacement polymorphism (Table 3). At the 5′ α-globin gene, ten of the 21 nonsynonymous mutations exhibited significant allele frequency differences between high- and low-altitude samples, and at six of these sites, the derived mutation was present at a frequency greater than 0.10 in the high-altitude sample. At the 3′ α-globin gene, 21 of the 33 nonsynonymous mutations exhibited significant allele frequency differences between high- and low-altitude samples, and at eight of these sites, the derived mutation was present at a frequency greater than 0.10 in the high-altitude sample (Table S1). This pattern of polymorphism is consistent with a model of diversifying selection that favors different protein variants in different elevational zones [9,21].

**Table 3:**
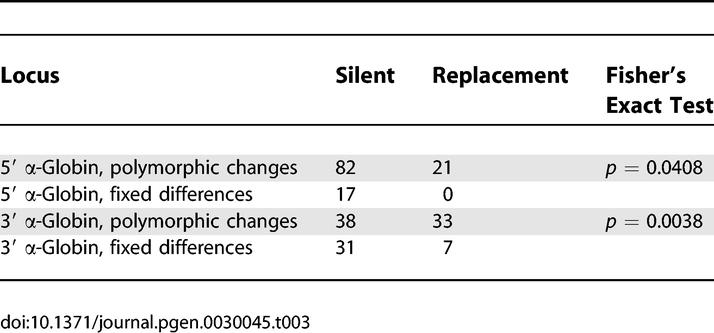
McDonald-Kreitman Neutrality Tests

### Linkage Disequilibrium and Altitudinal Differentiation

In contrast to patterns of nucleotide variation at five other unlinked nuclear loci, both α-globin paralogs exhibited a highly significant excess of intragenic linkage disequilibrium as revealed by Kelly's *Z*_ns_ statistic [34] (Table 4). This excess linkage disequilibrium, in combination with the extremely high levels of nucleotide diversity at both genes, is consistent with a model of diversifying selection in which the maintenance of balanced polymorphism produces nonrandom associations between alleles that are specific to different haplotype backgrounds [35,36]. In addition to high levels of linkage disequilibrium within both α-globin paralogs, intergenic comparisons of amino acid replacement polymorphism revealed a highly significant level of genotypic linkage disequilibrium between the two paralogs (*p* < 0.001). This result is consistent with the pattern of two-locus linkage disequilibrium documented in previous electrophoretic surveys [8,14,15,21].

**Table 4:**
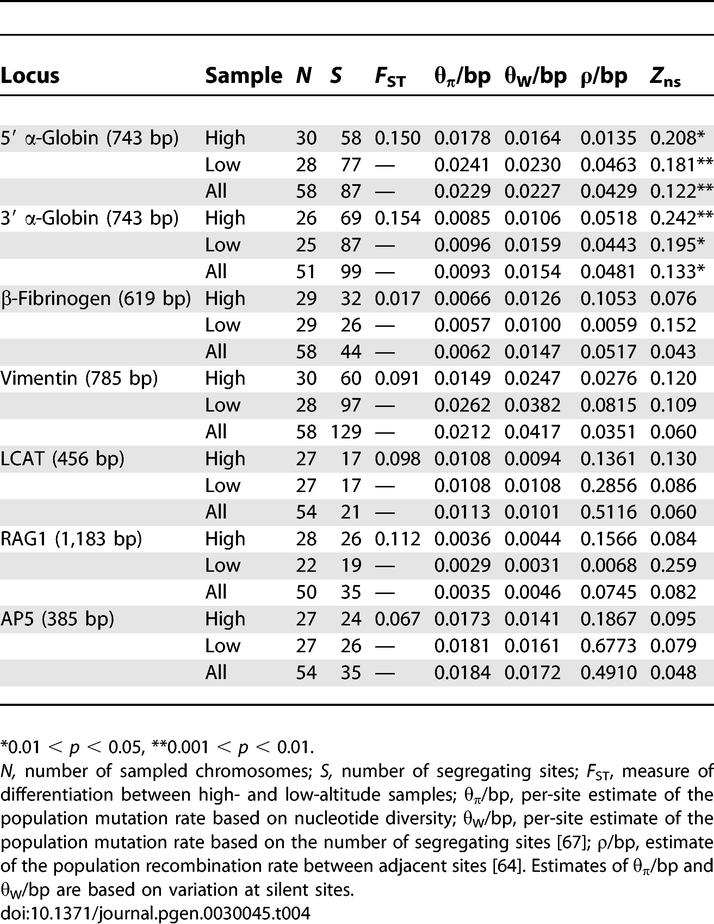
DNA Sequence Variation at Nuclear Genes of Deer Mice Sampled from a Pair of High- and Low-Altitude Localities in Colorado (Mt. Evans and Yuma County, Respectively)

In principle, it should be possible to determine whether a given polymorphism is subject to divergent selection between different environments by comparing the locus-specific level of differentiation to a multilocus average for unlinked neutral markers [37,38]. Again, consistent with a model of diversifying selection between different elevational zones, both α-globin genes are characterized by higher levels of altitudinal differentiation than five other unlinked nuclear loci (Table 4). However, it is important to recognize that point estimates of genetic differentiation have a large variance, which stems primarily from stochastic variance among the genealogies of unlinked loci. To account for this source of variance, we used coalescent simulations to generate a null distribution of site-specific *F*_ST_ values under a neutral model of population structure (see Materials and Methods). For each of the two α-globin genes, the null distribution was then used to identify specific replacement polymorphisms that exhibit levels of altitudinal differentiation that are too high to be explained by drift alone.

**Figure 2:**
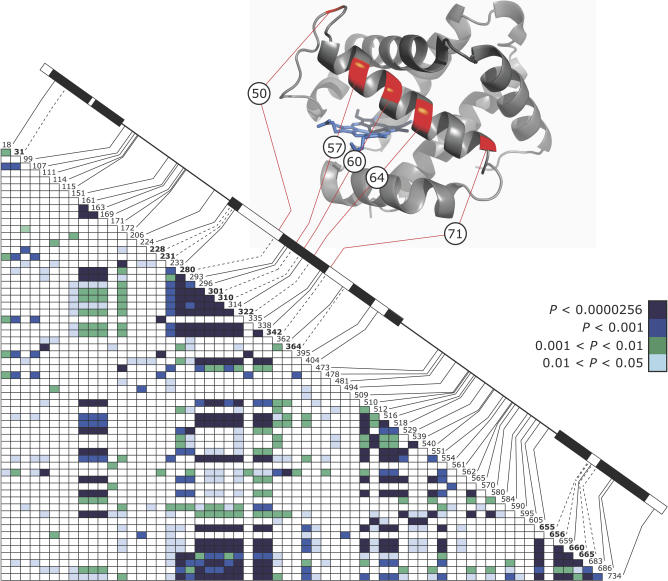
Plot of Linkage Disequilibrium across the 5′ α-Globin Gene, Showing Pairwise Associations between Informative Nucleotide Polymorphisms in the Total Sample of 74 Chromosomes In the diagram of α-globin gene structure, the three exons are depicted as boxes, the exonic regions that encode the six α helices (A, B, E, F, G, and H) are shown in black, and nonsynonymous polymorphisms are denoted by dotted lines. In the triangle matrix, *p*-values from Fisher's exact test are given for each pairwise comparison of informative polymorphisms (the Bonferroni-adjusted α level is 0.0000256). Amino acid polymorphisms at residue positions (CD15)50, (E6)57, (E9)60, (E9)64, (EF1)71, and (GH4)116 (mentioned in the text) are attributable to nonsynonymous mutations at nucleotide positions 280, 301, 310, 322, 342, and 665, respectively.

If high blood oxygen affinity represents a derived condition for high-altitude deer mice, then elevated levels of altitudinal differentiation at specific α-globin polymorphisms should be attributable to the increased frequency of derived mutations in the high-altitude population. Thus, the best candidate sites for divergent selection on hemoglobin function are those that are characterized by an unusually high *F*_ST_ value and an unusually high frequency of the derived allele at high altitude. At the 5′ α-globin gene, replacement polymorphisms at five closely linked residue positions, 50(CD15)His/Pro, 57(E6)Gly/Ala, 60(E9)Ala/Gly, 64(E13)Asp/Gly, and 71(EF1)Gly/Ser, and one more distant position, 116(GH4)Glu/Asp, were characterized by the highest site-specific *F*_ST_ values in comparisons between the high-altitude sample (Mt. Evans, Colorado, United States) and each of the two low-altitude samples (Yuma County, Colorado, and Pawnee County, Kansas, United States), and site-specific levels of altitudinal differentiation exceeded neutral expectations in both pairwise comparisons (Figure S1A and S1B). The replacement polymorphisms at sites 50, 57, 60, 64, and 71 are in highly significant linkage disequilibrium with one another (*p* < 0.00001 in all pairwise comparisons) and with site 116 (*p* < 0.025 in all pairwise comparisons; Figure 2). These nonrandom associations are not solely attributable to reduced recombination, as pairwise linkage disequilibria decay to background levels over a distance of less than 600 bp across the gene (Figure S2). The fact that two-locus α-globin haplotypes are maintained despite this short-range decay in linkage disequilibrium is consistent with the hypothesis of Chappell et al. [13] that coadapted combinations of protein alleles at the two genes are maintained by epistatic selection.

**Figure 3:**
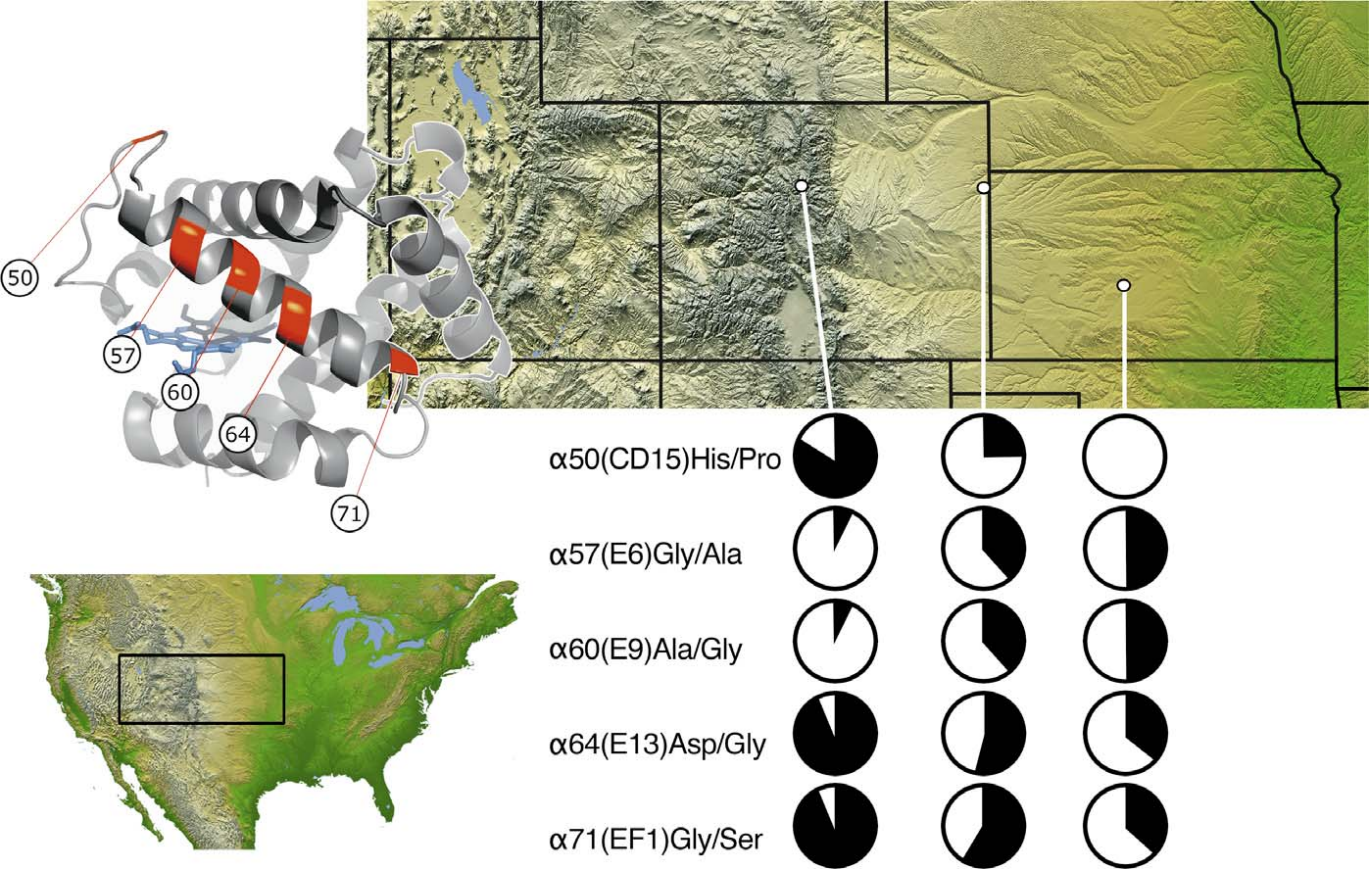
Altitudinal Patterns of Allele Frequency Variation at Five Amino Acid Replacement Polymorphisms in the 5′ α-Globin Gene that Span the E-Helix Domain of the Encoded Polypeptide In the pie diagram for each polymorphic site, the frequency of the derived allele is shown in black. From west to east, the sampling localities are 1, Mt. Evans, Clear Creek County, Colorado (4,347 m); 2, Bonny Reservoir, Yuma County, Colorado (1,158 m); and 3, Fort Larned National Monument, Pawnee County, Kansas (620 m).

In the case of sites 50, 64, and 71, the derived variants are present at unusually high frequency in the high-altitude sample (Table S1) and exhibit a monotonic increase in frequency as a positive function of altitude (Figure 3). Moreover, high levels of differentiation at the five closely linked replacement polymorphisms (sites 50, 57, 60, 64, and 71) and the replacement polymorphism at site 116 are each associated with elevated *F*_ST_ values at closely linked silent sites (Figure S1). Because the simulation results suggest that observed levels of altitudinal differentiation at these six replacement polymorphisms are too high to be explained by a neutral model of population structure, the elevated *F*_ST_ values at closely linked silent sites may be attributable to genetic hitchhiking [39,40].

**Figure 4:**
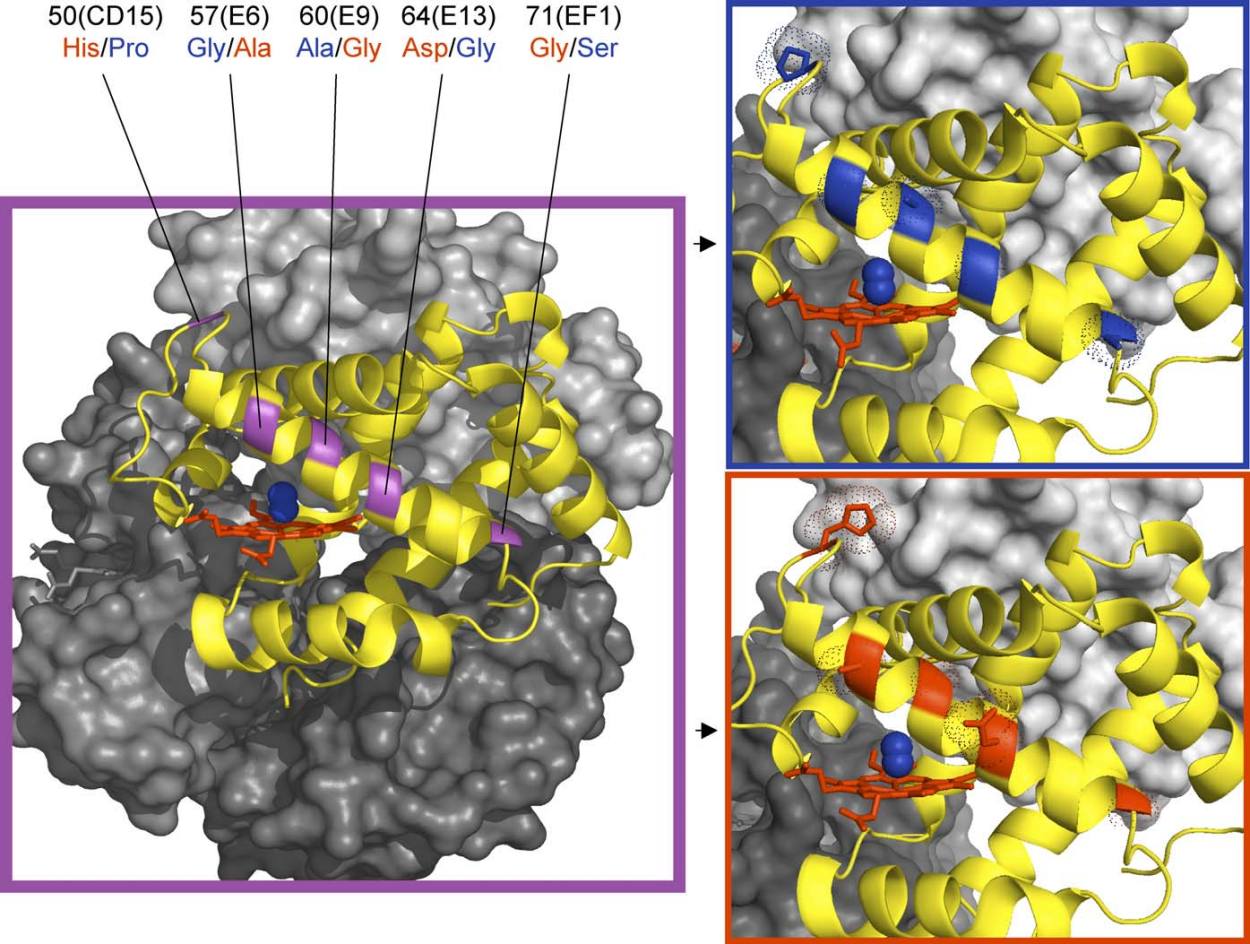
Homology-Based Structural Model of P. maniculatus Oxyhemoglobin, Showing the Location of Five Amino Acid Replacement Polymorphisms in the 5′ α-Globin Gene that Span the E-Helix Domain of the Encoded α-Chain Polypeptide The heme group (ferroprotoporphyrin IX) of the α-chain is shown in red, and the bound O_2_ molecule is shown in blue. Top right: The five amino acid variants that predominate in the high-altitude sample (50Pro/57Gly/60Ala/64Gly/71Ser) are shown in blue. Bottom right: The five amino acid variants that predominate in the low-altitude samples (50His/57Asp/60Gly/64Asp/71Gly) are shown in red.

Of the six replacement polymorphisms in 5′ α-globin that exhibit higher-than-expected *F*_ST_ values, the 64(E13)Asp/Gly polymorphism is predicted to have the most important functional consequences with respect to oxygen-binding kinetics of the hemoglobin protein. At site 64(E13), substitution of the uncharged glycine residue for the negatively charged aspartic acid residue involves the substitution of a single H atom side-chain for a much larger CH_2_-COOH side chain (Figure 4). As a result of the charge and size differences between these two amino acid residues, this 64(E13)Asp→Gly substitution reduces steric hindrance to oxygen binding in the heme pocket and is predicted to produce a substantial increase in oxygen-binding affinity (Table 5). The predicted effect of this mutation is corroborated by functional studies of the same 64(E13)Asp→Gly mutation in human hemoglobin. Functional studies revealed that the rare hemoglobin variant defined by the 64(E13)Asp→Gly mutation, “Hemoglobin Guangzhou-Hangzhou,” is characterized by an increased oxygen-binding affinity [41,42].

**Table 5:**
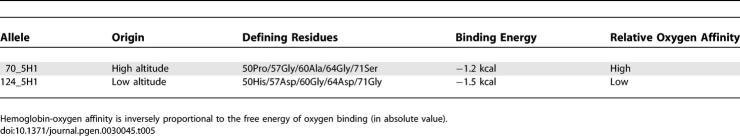
Predicted Oxygen Binding Energies of 5′ α-Globin Protein Alleles That Are Representative of High- and Low-Altitude Samples

In summary, the erythrocytes of deer mice contain an extraordinary diversity of functionally distinct hemoglobin isoforms due to variation between duplicated α-globin genes in addition to allelic variation that is segregating at each of the two genes. We suggest that functionally important mutations that distinguish the two α-globin paralogs, such as the 58(E7)His→Gln substitution in the 3′ α-globin gene, are primarily involved in maintaining a physiological division of labor between 5′ and 3′ α-chain hemoglobin tetramers and may therefore provide a regulatory reserve of oxygen transport capacity. By contrast, functionally important mutations that distinguish high- and low-altitude alleles, such as the 64(E13)Asp/Gly polymorphism in the 5′ α-globin gene, are primarily involved in adaptation of hemoglobin function to different elevational zones.

### Evidence for the Adaptive Maintenance of Balanced Polymorphism

**Figure 5:**
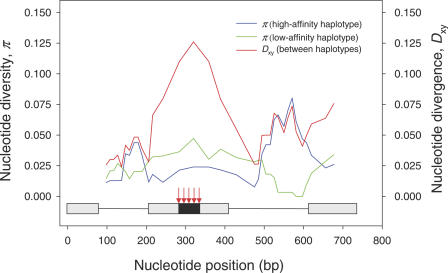
Sliding Window Plot Showing Variation in Levels of Silent Site Diversity within and between Functionally Defined Haplotype Classes of the 5′ α-Globin Gene In comparisons between the high- and low-affinity protein haplotypes, a pronounced peak of silent site divergence is directly centered on the region of exon 2 that harbors the five class-defining replacement polymorphisms (indicated by red arrows). The region of exon 2 that encodes the E-helix domain (nucleotides 285 through 335) is shown in black. The protein haplotype with high oxygen-binding affinity (50Pro/57Gly/60Ala/64Gly/71Ser) predominates in the high-altitude sample, whereas the protein haplotype with low oxygen-binding affinity (50His/57Asp/60Gly/64Asp/71Gly) predominates at low altitude.

When two or more functionally distinct alleles are maintained by some form of balancing selection, such as diversifying selection that favors different genotypes in spatially separated habitats, coalescence times for sequences belonging to different selectively defined allele classes are expected to be much longer than those for sequences belonging to the same allele class [35,36,40,43,44]. Consequently, comparisons of sequence variation between different allele classes should reveal a pronounced peak in nucleotide divergence that is centered on the selected site that defines the different classes. To determine whether the two α-globin paralogs exhibit this characteristic signature of diversifying selection, we conducted a sliding-window analysis of silent site diversity in comparisons between functionally distinct allele classes of each α-globin gene. Silent-site diversity within each allele class was estimated by π [45], and silent-site divergence between allele classes was estimated by *D*_xy_, the average number of nucleotide substitutions per site (Equation 12.65 in Nei and Kumar [45]). In the case of 5′ α-globin, the sliding-window analysis revealed a pronounced peak of silent site divergence in comparisons between alleles defined by the five closely linked replacement polymorphisms that span the E-helix domain of the encoded polypeptide (sites 50, 57, 60, 64, and 71; Figure 5). This peak of silent site divergence between the two allele classes is directly centered on the region of exon 2 that harbors all five class-defining replacement polymorphisms, as predicted if one or more of these sites represents the target of divergent selection [35,40,43]. In the window spanning this region of exon 2, silent site divergence between the two allele classes is 0.121 (Jukes-Cantor corrected), a value that exceeds estimates of average silent site divergence between the 5′ α-globin orthologs of P. maniculatus and P. leucopus (Table 2). This extraordinarily high level of sequence divergence between the two allele classes is consistent with the maintenance of two functionally divergent protein alleles as a long-term balanced polymorphism.

Based on the deduced isoelectric points of the translated amino acid sequences, the five-site protein haplotype that predominates at high altitude (50Pro/57Gly/60Ala/64Gly/71Ser) and the alternative haplotype that predominates at low altitude (50His/57Asp/60Gly/64Asp/71Gly) are referable to the *Hba* ^0^ and *Hba* ^1^ alleles, respectively, of Snyder et al. [21]. The predicted rank-order of oxygen-binding affinities for these alleles (Table 5) is also consistent with previous experimental results [10,12,13]. On the basis of previous physiological experiments [12,13], the “high affinity” protein haplotype is expected to confer enhanced physiological performance under hypoxic conditions at high altitude, whereas the alternative “low affinity” haplotype is expected to confer enhanced physiological performance at low altitude. The fact that the 5′ α-globin gene exhibits such a clear signature of diversifying selection is consistent with the idea that protein alleles with different oxygen-binding affinities are favored in different elevational zones [9,10,12,13,21].

Because hemoglobins from different species are typically distinguished by multiple amino acid substitutions, only a fraction of which may be functionally significant, it may often be difficult to identify the causal variants that are directly involved in molecular adaptation to different environments. Species like P. maniculatus provide the opportunity to pinpoint the specific amino acid changes that are involved in hemoglobin adaptation because it is possible to compare the sequences of functionally distinct alleles that are distinguished by a relatively small number of mutations. Adaptive modifications of hemoglobin function are generally thought to involve key changes in heme–protein contacts [such as the 58(E7)His→Gln substitution that distinguishes the two α-globin paralogs], intersubunit contacts, or binding sites for heterotropic ligands [17–20]. In contrast to this prevailing view, sequence variation at 5′ α-globin reveals that the enhanced oxygen affinity of the high-altitude protein allele is primarily attributable to a charge-changing 64(E13)Asp→Gly mutation on the exterior of the E-helix that reduces steric hindrance to oxygen binding in the heme pocket. Results of this study therefore reveal an unexpected mechanism underlying allelic differences in hemoglobin-oxygen affinity.

The population genetic analysis of α-globin polymorphism also provides an opportunity to assess the possible adaptive significance of epistasis among different mutations within the same gene. For example, in the case of 5′ α-globin, the derived glycine mutant at site 64(E13) is in nearly perfect linkage disequilibrium with the derived proline mutant at site 50(CD15). The latter residue is located in an exterior, interhelical segment of the α-globin polypeptide and therefore has no direct effect on ligand binding. However, it is worth noting that the change from a positively charged histidine residue to an uncharged proline residue at site 50(CD15) exactly offsets the change from a negatively charged aspartic acid residue to an uncharged glycine residue at site 64(E13). This suggests the hypothesis that the 64(E13)Asp→Gly mutation represents the primary adaptive change that produced the enhanced hemoglobin-oxygen affinity and that the 50(CD15)His→Pro mutation represents a compensatory change that maintains the same net surface charge of the molecule. Functional experiments involving site-directed mutagenesis will be required to measure the independent and joint effects of these mutations on hemoglobin-oxygen affinity. In addition to the possible role of epistasis among mutations in the same gene, further work is also needed to assess the potential for epistatic interactions among genes such as the α- and β-globin loci that encode interacting subunits of the same tetrameric protein.

## Materials and Methods

### Samples.

Mice were live-trapped and handled in accordance with Institutional Animal Care and Use Committee guidelines. Sample sizes, sample localities, and specimen numbers are as follows: summit of Mt. Evans, Clear Creek County, Colorado (4,347 m; *n* = 15, JFS 70, 71, 73, 74, 76, 80 to 82, 87, 88, 95, 97, 98, 100, and 106), Bonny Reservoir, Yuma County, Colorado (1,158 m; *n* = 15, JFS 107, 114, 117, 122 to 124, 132, 134 to 137, and 140 to 143), and Fort Larned National Monument, Pawnee County, Kansas (620 m; *n* = 11, NK 53239, 53245, 53247 to 53249, 53251, 53252, 53256, 53265, 53266, and 53268). After each mouse was killed, whole blood was collected by cardiac puncture (in the case of mice collected from Mt. Evans and Yuma County) and liver tissue was frozen in liquid nitrogen as a source of genomic DNA. Specimens were deposited in the vertebrate collection at the University of Nebraska State Museum and the Museum of Southwestern Biology, University of New Mexico. DNA was extracted from frozen liver of each mouse using DNeasy kits (Qiagen, http://www.qiagen.com).

### Cloning and sequencing globin genes.

Adult α-globin genes are duplicated in nearly all mammals, including *Peromyscus* [8,31,46]. Thus, before conducting surveys of population-level variation at individual α-globin genes, it was first necessary to characterize the genomic structure of the α-globin gene family in order to design locus-specific PCR primers that would not coamplify paralogous gene duplicates or pseudogenes. The use of locus-specific primers is critical because it allows us to distinguish between allelic variation segregating at a single gene versus variation between duplicated genes. To isolate and characterize the α-globin genes of P. maniculatus we screened a bacteriophage-λ genomic library using the lambda FIX II/XhoI partial fill-in vector kit (Stratagene, http://www.stratagene.com). We successfully cloned one of the two α-globin paralogs (3′ α-globin) in a single fragment encompassing approximately 16.5 kb of *Peromyscus* Chromosome 13, which is syntenic to *Mus* Chromosome 11. We then used degenerate primers from the 5′ and 3′ flanking regions to amplify and sequence the other paralog (5′ α-globin). The design of locus-specific primers allowed us to amplify and sequence approximately 900-bp fragments that contain the entire coding sequence of each of the separate α-globin paralogs.

In order to resolve the haplotype phase of each α-globin paralog (i.e., to associate nucleotides at multiple heterozygous sites), we adopted an experimental protocol that involved cloning both alleles of diploid PCR products in all individuals. After cloning diploid PCR products into pCR4-TOPO vector (Invitrogen, http://www.invitrogen.com), we sequenced the complete coding region and intervening introns of at least 16 cloned alleles per mouse (at least eight cloned alleles per paralog). This labor-intensive strategy offered two important advantages: (1) we recovered diploid genotypes for the complete coding region of both α-globin paralogs, and (2) we recovered the exact haplotype phase of all heterozygous sites.

We also obtained a representative sequence of the β-globin gene of P. maniculatus in order to construct an accurate homology-based structural model of the hemoglobin tetramer. Primers for β-globin were designed using an alignment of orthologous sequences from human, *Rattus,* and *Mus,* in addition to published sequence from part of the β-globin coding region in *Peromyscus* [47]. We then used a genome-walking approach to characterize the 5′ and 3′ flanking sequence, and this enabled us to design primers that amplified the complete coding region.

The globin genes were PCR-amplified using Ampli-taq Gold chemistry (Applied Biosystems, http://www.appliedbiosystems.com) under the following cycling parameters: 94 °C (120 s) initial denaturing, [94 °C (30 s), 58 °C (30 s), 72 °C (60 s)] 30 times, and a final extension at 72 °C (120 s). Primer sequences are listed in Table S2. The same PCR primers were also used for sequencing reactions, although annealing temperature was increased to 60 °C. M13 primers were used to amplify cloned products (55 °C annealing temperature) and internal T7/T3 primers were used for sequencing. All sequencing reactions and samples were run on an ABI 3730 capillary sequencer using Dye Terminator chemistry (Applied Biosystems). For each of the two α-globin gene duplicates, we used orthologous sequence from a related species, *P. leucopus,* to estimate divergence and to infer the ancestral and derived variants at each segregating site in P. maniculatus.

### Unlinked nuclear loci.

For the purpose of comparison with the α-globin genes, we used a sample of 30 mice from Mt. Evans and Yuma County to survey DNA sequence variation at five additional unlinked nuclear loci: β-fibrinogen (614 bp), vimentin (785 bp), LCAT (456 bp), RAG1 (1,183 bp), and AP5 (385 bp). Each of the five loci were PCR-amplified as described above and were then sequenced directly on the ABI 3730. Primers and sequenced gene regions for each marker locus are listed in Table S2. After first obtaining direct sequence data for the complete sample of 30 mice, we cloned both alleles from greater than 80% of individuals that were heterozygous at multiple sites. We then used the program PHASE [48,49] to infer haplotype phase for the remaining individuals. We found that by experimentally verifying haplotype phase for large numbers of multiple heterozygotes, the PHASE program was able to resolve the remaining haplotypes with a high level of confidence.

### IEF analysis of protein variation.

The original experiments that documented the association between physiological phenotype and α-globin genotype in P. maniculatus were based on gel electrophoresis and thin-layer IEF analysis of protein variation [8–13]. In order to relate the results of our DNA sequence analysis to this previously published work, we conducted an immobilized gradient, thin-layer IEF analysis to obtain electrophoretic protein genotypes for the same mice that we used in the analysis of DNA sequence variation. By matching deduced amino acid sequences of each allele to the observed IEF genotypes, we identified the specific amino acid changes that underlie differences in isoelectric point between the protein alleles of 5′ α-globin (the *Hba* ^0^ and *Hba* ^1^ alleles of Snyder et al. [21]). Hemolysates and globin samples were prepared following Ferrand [50,51], and thin-layer IEF was carried out in polyacrylamide gels (pH 7.1 to 8.1).

### Modeling of structure–function relationships in deer mouse hemoglobin.

The primary structures of the α- and β-globin polypeptides of P. maniculatus were deduced from translated DNA sequences (Figure 1). To construct a homology-based model of deer mouse hemoglobin, we used the P. maniculatus amino acid sequences to search structural templates on the 3D-JIGSAW [52] and SWISS-MODEL [53] Web servers. The resulting homology models were evaluated using the Procheck protein crystallography program from the CCP4 suite [54]. All structural mining analyses, including superimpositions and calculation of interatomic distances, were performed using Swiss-Pdb Viewer [55]. Calculations of local binding energy minimization were conducted using AutoDock [56]. All graphical representations of molecular structures were prepared using PyMOL (DeLano Scientific, http://www.delanoscientific.com).

### Genetic data analysis.

DNA sequences were aligned and contigs were assembled using the Sequencher program (Gene Codes, http://www.genecodes.com). All sequences were verified by visual inspection of chromatograms. To detect evidence of gene conversion (nonreciprocal recombination) between the two α-globin paralogs, we used a maximum likelihood algorithm to estimate ψ, the per-site probability of detecting a gene conversion event between the two paralogous sequences [57]. To detect evidence of recombination within each of the two α-globin paralogs, we used the four-gamete test of Hudson and Kaplan [58] to estimate *R*_M_, the minimum number of recombination events in the history of the sample, and we used coalescent simulations to obtain 95% confidence intervals for the estimates.

For each pairwise combination of nonsynonymous nucleotide polymorphisms, we tested the null hypothesis that genotypes at one site were independent of genotypes at a second site by performing exact tests on contingency tables of diploid genotypes. These tests were performed for site-by-site comparisons between the two α-globin paralogs. To obtain a summary measure of two-locus linkage disequilibrium between the two α-globin paralogs, we used the index of association, *I*_A_ [59,60], and tested for significant associations using the randomization test of Agapow and Burt [61]. In the randomization tests, each of the two α-globin paralogs were treated as separate linkage groups and permutations of diploid genotypes were restricted to each group. Thus, excess linkage disequilibrium in the observed data relative to the randomized data can be attributed to nonrandom associations between the two paralogs. Likewise, permutations of genotypes within each linkage group were restricted to each of the separate population samples. Thus, excess linkage disequilibrium in the actual data relative to the randomized data cannot be solely attributed to genetic differentiation between the high- and low-altitude samples. Finally, the identified gene conversion tracts were treated as missing data and were fixed in place for the randomization tests, thereby ensuring that the actual distribution of missing data was preserved in the randomized datasets.

Summary statistics of nucleotide polymorphism and divergence were computed with the programs SITES [62] and DnaSP v4.0 [63]. To assess whether the observed levels of intralocus linkage disequilibrium deviated from neutral expectations, we conducted neutrality tests based on Kelly's *Z*_ns_ statistic [34]. To obtain critical values for this test statistic, we generated a null distribution of *Z*_ns_ values by using coalescent simulations to generate 10,000 neutral genealogies that were conditioned on estimates of ρ (= 4*Nc*, where *N* is the effective population size and *c* is the rate of crossing over between adjacent sites) [64]. To measure levels of genetic differentiation between high- and low-altitude samples, we calculated Weir and Cockerham's [65] estimator of *F*_ST_ for each single nucleotide polymorphism, and we used the estimator of Hudson et al. [66] to compute an analogous measure of differentiation for complete sequence haplotypes. To determine whether amino acid polymorphisms in the α-globin genes exhibit unusually high levels of altitudinal differentiation that may be attributable to diversifying selection, we used coalescent simulations to generate null distributions of site-specific *F*_ST_ values under a neutral model of population structure. Specifically, we used observed levels of differentiation at silent-sites to parameterize a 100-deme island model of population structure in which the simulated migration rate between each pair of sampled demes was set equal to the value that produced the observed mean *F*_ST_ value. For each α-globin gene, we used coalescent simulations to generate 10,000 neutral genealogies that were conditioned on the actual sample size and estimates of *θ* (= 4*N*μ, where *N* is the effective population size and μ is the mutation rate) [67]. Separate distributions of *F*_ST_ values were generated for a sliding window of 50 bp that was positioned at 10-bp increments along each of the two α-globin genes.

## Supporting Information

Figure S1 Variation in Site-Specific Levels of Altitudinal Differentiation across the 5′ α-Globin Gene of P. maniculatus
(A) Comparison between high- and low-altitude samples (Mt. Evans, Colorado [4,347 m] versus Pawnee County, Kansas [620 m]).
(B) Comparison between high- and low-altitude samples (Mt. Evans versus Yuma County, Colorado [1,158 m]).
(C) Comparison between the two low-altitude localities (Pawnee County versus Yuma County).
Open diamonds denote *F*_ST_ values for nonsynonymous nucleotide polymorphisms (*n* = 21 sites). The red line represents a sliding-window plot of variation in site-specific *F*_ST_ values for synonymous and noncoding nucleotide polymorphisms across the gene. Red asterisks mark the mid-point of 100-bp windows containing one or more replacement polymorphisms that exhibited higher-than-expected *F*_ST_ values.
(13 KB PDF)

Figure S2 Relationship between Pairwise Linkage Disequilibrium and Distance in bp Filled symbols denote 201 pairwise associations that were significant by a Fisher's exact test after Bonferroni correction
(128 KB PDF)

Table S1 Amino Acid Variation in the α-Globin Genes of High- and Low-Altitude Deer Mice (80 KB DOC)

Table S2 Additional Information on Sequenced Loci (64 KB DOC)

### Accession Numbers

The GenBank (http://www.ncbi.nlm.nih.gov/Genbank) accession numbers for all sequences discussed in this paper are EF369525–EF370032.

## Abbreviation

bp: base pair(s)
IEF: isoelectric focusing

## References

[pgen-0030045-b001] Nachman MW, Hoekstra HE, D'Agostino SL (2003). The genetic basis of adaptive melanism in pocket mice. Proc Natl Acad Sci U S A.

[pgen-0030045-b002] Wheat CW, Watt WB, Pollock DD, Schulte PM (2006). From DNA to fitness differences: Sequences and structures of adaptive variants of *Colias* phosphoglucose isomerase (PGI). Mol Biol Evol.

[pgen-0030045-b003] Gillespie JH (1991). The causes of molecular evolution.

[pgen-0030045-b004] Feder ME, Watt WB, Berry RJ, Crawford TJ, Hewitt GM (1992). Functional biology of adaptation. Genes in ecology.

[pgen-0030045-b005] Mitton JB (1997). Selection in natural populations.

[pgen-0030045-b006] Eanes WF (1999). Analysis of selection on enzyme polymorphisms. Annu Rev Ecol Syst.

[pgen-0030045-b007] Watt WB, Dean AM (2000). Molecular-functional studies of adaptive genetic variation in prokaryotes and eukaryotes. Annu Rev Genet.

[pgen-0030045-b008] Snyder LRG (1980). Closely linked alpha-chain hemoglobin loci in Peromyscus maniculatus and other animals: Speculations on the evolution of duplicate loci. Evolution.

[pgen-0030045-b009] Snyder LRG (1981). Deer mouse hemoglobins: Is there genetic adaptation to high altitude?. Bioscience.

[pgen-0030045-b010] Snyder LRG (1985). Low P_50_ in deer mice native to high altitude. J Appl Physiol.

[pgen-0030045-b011] Snyder LRG, Born S, Lechner AJ (1982). Blood oxygen affinity in high- and low-altitude populations of the deer mouse. Respir Physiol.

[pgen-0030045-b012] Chappell MA, Snyder LRG (1984). Biochemical and physiological correlates of deer mouse α chain hemoglobin polymorphisms. Proc Natl Acad Sci U S A.

[pgen-0030045-b013] Chappell MA, Hayes JP, Snyder LRG (1988). Hemoglobin polymorphisms in deer mice *(Peromyscus maniculatus):* Physiology of beta-globin variants and alpha-globin recombinants. Evolution.

[pgen-0030045-b014] Snyder LRG (1978). Genetics of hemoglobin in the deer mouse, Peromyscus maniculatus. I. Multiple α- and β-globin structural loci. Genetics.

[pgen-0030045-b015] Snyder LRG (1978). Genetics of hemoglobin in the deer mouse, Peromyscus maniculatus. II. Multiple alleles at regulatory loci. Genetics.

[pgen-0030045-b016] Hayes JP, O'Connor CS (1999). Natural selection on thermogenic capacity of high-altitude deer mice. Evolution.

[pgen-0030045-b017] Perutz MF (1983). Species adaptation in a protein molecule. Mol Biol Evol.

[pgen-0030045-b018] Poyart C, Wajcman H, Kister J (1992). Molecular adaptation of hemoglobin-function in mammals. Respir Physiol.

[pgen-0030045-b019] Weber RE, Fago A (2004). Functional adaptation and its molecular basis in vertebrate hemoglobins, neuroglobins and cytoglobins. Respir Physiol Neurobiol.

[pgen-0030045-b020] Storz JF (2007). Hemoglobin function and physiological adaptation to hypoxia in high-altitude mammals. J Mammal.

[pgen-0030045-b021] Snyder LRG, Chappell MA, Hayes JP (1988). α-Chain hemoglobin polymorphisms are correlated with altitude in the deer mouse, Peromyscus maniculatus. Evolution.

[pgen-0030045-b022] Shaanan B (1980). The crystal structure of oxyhaemoglobin at 2.1 A resolution. J Mol Biol.

[pgen-0030045-b023] Phillips SEV, Schoenborn B (1981). Neutron diffraction reveals oxygen-histidine hydrogen bond in oxymyoglobin. Nature.

[pgen-0030045-b024] Perutz MF, Steinberg MH, Forget BG, Higgs DR, Nagel RL (2001). Molecular anatomy and physiology of hemoglobin. Disorders of hemoglobin: genetics, pathophysiology, and clinical management.

[pgen-0030045-b025] Nagai K, Luisi B, Shih D, Miyazaki G, Imai K (1987). Distal residues in the oxygen binding site of haemoglobin studied by protein engineering. Nature.

[pgen-0030045-b026] Weber RE, Lalthantluanga R, Braunitzer G (1988). Functional characterization of fetal and adult yak hemoglobins: An oxygen binding cascade and its molecular basis. Arch Biochem Biophys.

[pgen-0030045-b027] Hiebl I, Weber RE, Schneeganss D, Kosters J, Braunitzer G (1988). Structural adaptations in the major and minor hemoglobin components of adult Ruppell's griffon (Gyps ruepellii, Aegypiinae): A new molecular pattern for hypoxia tolerance. Biol Chem Hoppe-Seyler.

[pgen-0030045-b028] Hiebl I, Braunitzer G, Schneeganss D (1987). The primary sequence of the major and minor hemoglobin components of adult Andean goose (Chloephaga melanoptera, Anatidae): The mutation Leu-Ser in position 55 of the β chains. Biol Chem Hoppe-Seyler.

[pgen-0030045-b029] Hiebl I, Schneeganss D, Grimm F, Kosters J, Braunitzer G (1987). High altitude respiration in birds. The primary structures of the major and minor hemoglobin components of adult European black vulture (Aegypius monachus, Aegypiinae). Biol Chem Hoppe-Seyler.

[pgen-0030045-b030] Hiebl I, Weber RE, Schneeganss D, Braunitzer G (1989). The primary structure and functional properties of the major and minor hemoglobin components of the adult white-headed vulture (Trigonoceps occipitalis, Aegypiinae). Biol Chem Hoppe-Seyler.

[pgen-0030045-b031] Hardison R, Steinberg MH, Forget BG, Higgs DR, Nagel RL (2001). Organization, evolution and regulation of the globin genes. Disorders of hemoglobin: Genetics, pathophysiology, and clinical management.

[pgen-0030045-b032] Higgs DR, Vickers MA, Wilkie AOM, Pretorius I-M, Jarman AP (1989). A review of the molecular genetics of the human α-globin gene cluster. Blood.

[pgen-0030045-b033] McDonald JH, Kreitman M (1991). Adaptive protein evolution at the Adh locus in *Drosophila*. Nature.

[pgen-0030045-b034] Kelly JK (1997). A test of neutrality based on interlocus associations. Genetics.

[pgen-0030045-b035] Navarro A, Barton NH (2002). The effects of multilocus balancing selection on neutral variability. Genetics.

[pgen-0030045-b036] Strobeck C (1983). Expected linkage disequilibrium for a neutral locus linked to a chromosomal rearrangement. Genetics.

[pgen-0030045-b037] Beaumont MA (2005). Adaptation and speciation: What can *F*
_ST_ tell us?. Trends Ecol Evol.

[pgen-0030045-b038] Storz JF (2005). Using genome scans of DNA polymorphism to infer adaptive population divergence. Mol Ecol.

[pgen-0030045-b039] Charlesworth B, Nordborg M, Charlesworth D (1997). The effects of local selection, balanced polymorphism and background selection on equilibrium patterns of genetic diversity in subdivided populations. Genet Res.

[pgen-0030045-b040] Nordborg M, Innan H (2003). The genealogy of sequences containing multiple sites subject to strong selection in a subdivided population. Genetics.

[pgen-0030045-b041] Jen P, Liu Y (1987). Hemoglobin Guangzhou, alpha 64(E13)Asp-Gly, a new abnormal hemoglobin found in Guangzhou, China. Hemoglobin.

[pgen-0030045-b042] Zhou Z, Chen L, Chen P, Zhang K, Wang Y (1987). Hemoglobin Hangzhou alpha(E13)Asp-Gly. A new variant found in China. Hemoglobin.

[pgen-0030045-b043] Hudson RR, Kaplan NL (1988). The coalescent process in models with selection and recombination. Genetics.

[pgen-0030045-b044] Charlesworth D (2006). Balancing selection and its effects on sequences in nearby genome regions. PloS Genet.

[pgen-0030045-b045] Nei M, Kumar S (2000). Molecular evolution and phylogenetics.

[pgen-0030045-b046] Tufarelli C, Hardison R, Miller W, Hughes J, Clark K (2004). Comparative analysis of the alpha-like globin clusters in mouse, rat, and human chromosomes indicates a mechanism underlying breaks in conserved synteny. Genome Res.

[pgen-0030045-b047] Padgett RW, Loeb DD, Snyder LRG, Edgell MH, Hutchison CA (1987). The molecular organization of the beta-globin complex of the deer mouse, Peromyscus maniculatus. Mol Biol Evol.

[pgen-0030045-b048] Stephens M, Smith NJ, Donnelly P (2001). A new statistical method for haplotype reconstruction from population data. Am J Hum Genet.

[pgen-0030045-b049] Stephens M, Donnelly P (2003). A comparison of Bayesian methods for haplotype reconstruction from population genotype data. Am J Hum Genet.

[pgen-0030045-b050] Ferrand N (1990). Biochemical and genetic studies on rabbit hemoglobin. II. Electrophoretic polymorphism of the β-chain. Biochem Genet.

[pgen-0030045-b051] Ferrand N (1989). Biochemical and genetic studies on rabbit hemoglobin. I. Electrophoretic polymorphism of the β-chain. Biochem Genet.

[pgen-0030045-b052] Bates PA, Kelley LA, MacCallum RM, Sternberg MJE (2001). Enhancement of protein modeling by human intervention in applying the automatic programs 3D-JIGSAW and 3D-PSSM. Proteins Suppl.

[pgen-0030045-b053] Schwede T, Kopp J, Guex N, Peitsch MC (2003). SWISS-MODEL: An automated protein homology-modeling server. Nucleic Acids Res.

[pgen-0030045-b054] Collaborative Computational Project N (1994). The CCP4 Suite: Programs for protein crystallography. Acta Crystallogr D.

[pgen-0030045-b055] Guex N, Peitsch MC (1997). SWISS-MODEL and the Swiss-PdbViewer: An environment for comparative protein modeling. Electrophoresis.

[pgen-0030045-b056] Morris GM, Goodsell DS, Halliday RS, Huey R, Hart WE (1998). Automated docking using a Lamarckian genetic algorithm and an empirical binding free energy function. J Comp Chem.

[pgen-0030045-b057] Betran E, Rozas J, Navarro A, Barbadilla A (1997). The estimation of the number and the length distribution of gene conversion tracts from population DNA sequence data. Genetics.

[pgen-0030045-b058] Hudson RR, Kaplan NL (1985). Statistical properties of the number of recombination events in the history of a sample of DNA sequences. Genetics.

[pgen-0030045-b059] Brown AHD, Feldman MW, Nevo E (1980). Multilocus structure of natural populations of Hordeum spontaneum. Genetics.

[pgen-0030045-b060] Haubold B, Travisano M, Rainey PB, Hudson RR (1998). Detecting linkage disequilibrium in bacterial populations. Genetics.

[pgen-0030045-b061] Agapow PM, Burt A (2001). Indices of multilocus linkage disequilibrium. Mol Ecol Notes.

[pgen-0030045-b062] Hey J, Wakeley J (1997). A coalescent estimator of the population recombination rate. Genetics.

[pgen-0030045-b063] Rozas J, Sanchez-DelBarrio JC, Messeguer X, Rozas R (2003). DnaSP, DNA polymorphism analyses by the coalescent and other methods. Bioinformatics.

[pgen-0030045-b064] Hudson RR (1987). Estimating the recombination parameter of a finite population without selection. Genet Res.

[pgen-0030045-b065] Weir BS, Cockerham CC (1984). Estimating *F*-statistics for the analysis of population structure. Evolution.

[pgen-0030045-b066] Hudson RR, Boos DD, Kaplan NL (1992). A statistical test for detecting geographic subdivision. Mol Biol Evol.

[pgen-0030045-b067] Watterson GA (1975). On the number of segregating sites in genetical models without recombination. Theor Popul Biol.

