# Supplementary figures and images for: The Molecular Basis of High-Altitude Adaptation in Deer Mice
Source: PLoS Genet. 2007 Mar 30;3(3):e45. doi: 10.1371/journal.pgen.0030045 (PMC1839143; doi:10.1371/journal.pgen.0030045)

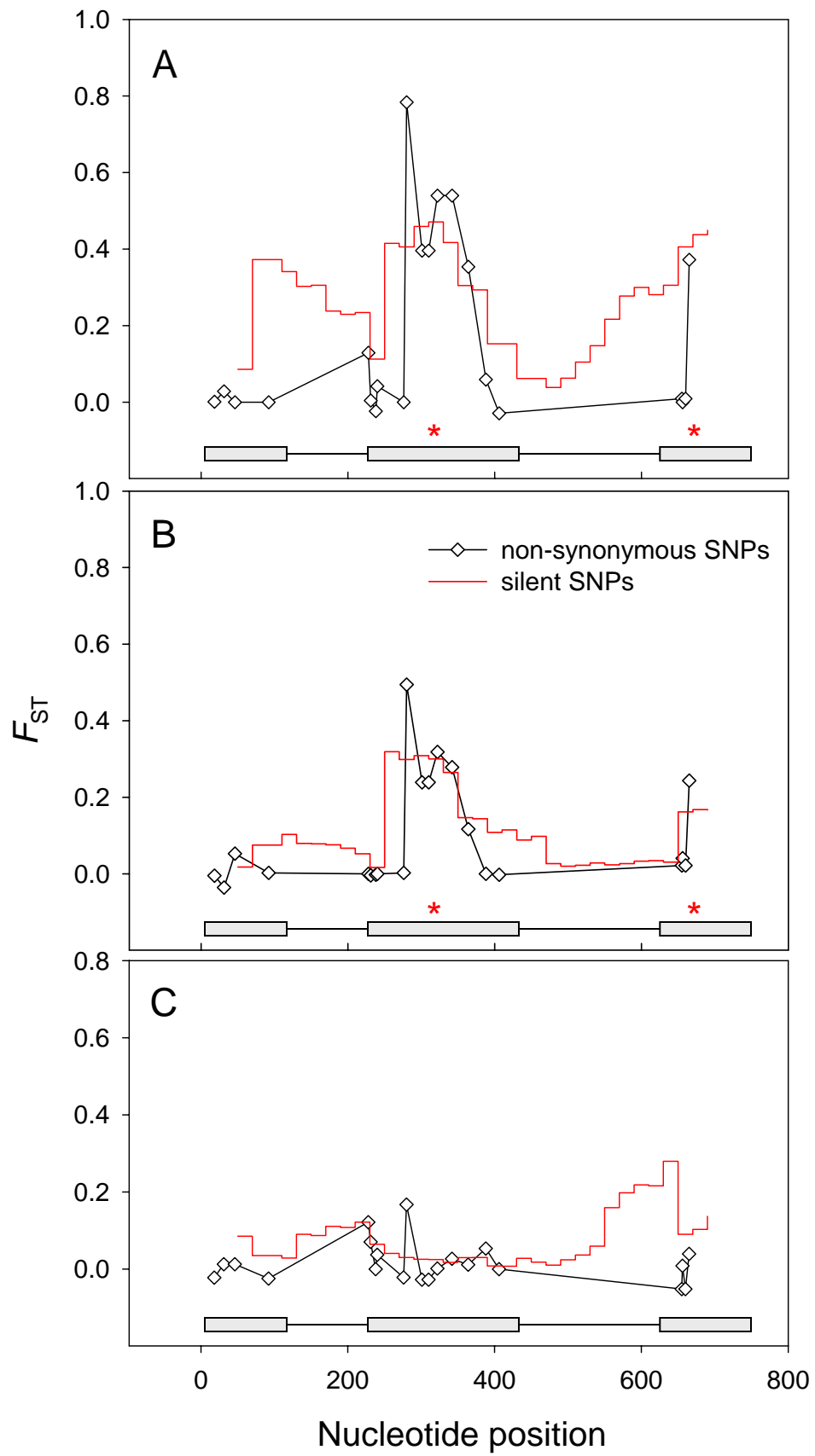

Supplement: Figure S1 — (A) Comparison between high- and low-altitude samples (Mt. Evans, Colorado [4,347 m] versus Pawnee County, Kansas [620 m]). (B) Comparison between high- and low-altitude samples (Mt. Evans versus Yuma County, Colorado [1,158 m]). (C) Comparison between the two low-altitude localities (Pawnee County versus Yuma County). Open diamonds denote F ST values for nonsynonymous nucleotide polymorphisms (n = 21 sites). The red line represents a sliding-window plot of variation in site-specific F ST values for synonymous and noncoding nucleotide polymorphisms across the gene. Red asterisks mark the mid-point of 100-bp windows containing one or more replacement polymorphisms that exhibited higher-than-expected F ST values. (13 KB PDF) [file pgen.0030045.sg001.pdf]

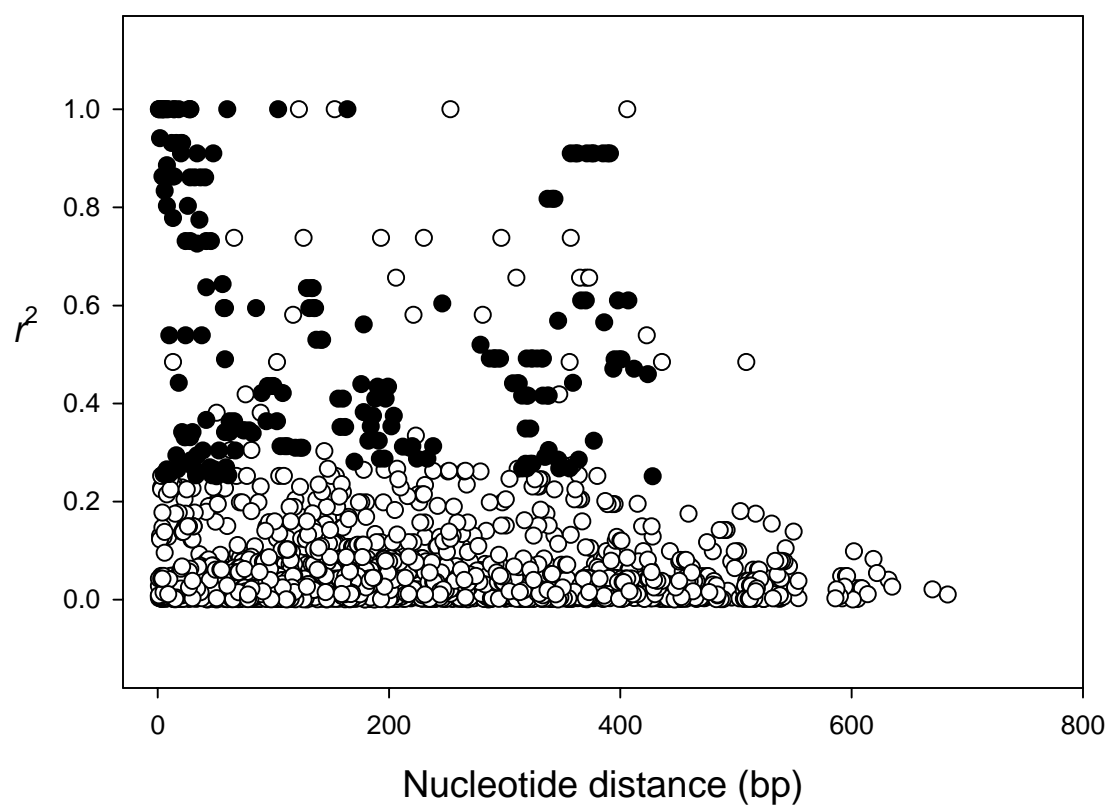

Supplement: Figure S2 — Filled symbols denote 201 pairwise associations that were significant by a Fisher's exact test after Bonferroni correction (128 KB PDF) [file pgen.0030045.sg002.pdf]
