# Supplementary material for: The Molecular Basis of High-Altitude Adaptation in Deer Mice
Source: PLoS Genet. 2007 Mar 30;3(3):e45. doi: 10.1371/journal.pgen.0030045 (PMC1839143; doi:10.1371/journal.pgen.0030045)
Supplement: Table S1 — (80 KB DOC) [file pgen.0030045.st001.doc]

Table S1. Amino acid replacement polymorphisms in deer mice where the derived variant is present at a frequency of >0.100 in the high-altitude sample.

| Nucleotide site | Codon change | Amino acid replacement^1^ | Frequency of derived variant at high-altitude | *F*_ST_ |  |  |
| --- | --- | --- | --- | --- | --- | --- |
|  |  |  |  | High vs. Low  (Mt. Evans vs. Pawnee Co.) | High vs. Low  (Mt. Evans vs. Yuma Co.) | Low vs. Low  (Yuma Co. vs. Pawnee Co.) |
| 5’ α-globin |  |  |  |  |  |  |
| 18 | GA**A**→GA**C** | 5(A3)Glu→Asp | 0.933 | 0.001 | -0.005 | -0.022 |
| 280 | C**A**C→C**C**C | 50(CD15)His→Pro | 0.833 | 0.784** | 0.494** | 0.167 |
| 322 | G**A**C→G**G**C | 64(E13)Asp→Gly | 0.933 | 0.540** | 0.318** | 0.002 |
| 342 | **G**GC→**A**GC | 71(EF1)Gly→Ser | 0.933 | 0.540** | 0.278** | 0.027 |
| 364 | G**G**T→G**C**T | 78(EF8)Gly→Ala | 1.000 | 0.354 | 0.117 | 0.011 |
| 665 | GA**G**→GA**T** | 116(GH4)Glu→Asp | 0.553 | 0.373* | 0.244* | 0.039 |
|  |  |  |  |  |  |  |
| 3’ α-globin |  |  |  |  |  |  |
| 7 | **C**TC→**T**TC  **G**TC→**T**TC | 2(NA2)Leu→Phe  2(NA2)Val→Phe | 0.115 | 0.127 | -0.005 | 0.089 |
| 18 | GA**G**→GA**C** | 5(A3)Glu→Asp | 0.231 | -0.035 | 0.057 | -0.019 |
| 37 | **A**CT→**G**CT | 12(A10)Thr→Ala | 0.115 | 0.013 | 0.081 | -0.042 |
| 59 | G**G**C→G**A**C | 19(AB1)Gly→Asp | 0.160 | 0.013 | -0.042 | -0.011 |
| 282 | **G**GC→**A**GC | 51(CD16)Gly→Ser | 0.115 | -0.031 | 0.246* | 0.124 |
| 364 | G**G**T→G**C**T | 78(EF8)Gly→Ala | 0.307 | 0.109 | 0.260* | 0.027 |
| 393 | **G**CT→**A**CT | 88(F9)Ala→Thr | 0.154 | 0.077 | 0.024 | -0.020 |
| 634 | C**T**G→C**A**G | 105(G12)Leu→Gln | 0.696 | 0.270 | 0.540** | 0.063 |

^1^For each polymorphism, the derived variant is shown on the right (e.g., for ‘Leu→Phe’, Leu is ancestral and Phe is derived).

**P*<0.05, ***P*<0.001 under a neutral model of population structure (see *Materials and Methods*).
