# Supplementary material for: The Molecular Basis of High-Altitude Adaptation in Deer Mice
Source: PLoS Genet. 2007 Mar 30;3(3):e45. doi: 10.1371/journal.pgen.0030045 (PMC1839143; doi:10.1371/journal.pgen.0030045)
Supplement: Table S2 — (64 KB DOC) [file pgen.0030045.st002.doc]

Table S2. Primer sequences for nuclear loci in *P. maniculatus*.

| Locus | Sequenced gene region | Length (bp) | Primers |  |
| --- | --- | --- | --- | --- |
| 5’ α-globin | Exons 1-3, introns 1-2 | 743 | D-1518 | 5’ CTTGCTCTGCAGCGCACC 3’ |
|  |  |  | D-2387R | 5’ CAAAGACCAAGAGGTACAG 3’ |
| 3’ α-globin | Exons 1-3, introns 1-2 | 743 | KEP107 | 5’ GGCCATGGTATGTCTCTAACTCC 3’ |
|  |  |  | KEP97 | 5’ CGTTAACACACTTCCTTGGGTC 3’ |
| β-globin | Exons 1-3, introns 1-2 | 1140 | BETA_PM_F1 | 5’ GTAGAGCAGGATCAGTTGC 3’ |
|  |  |  | BETA_PM_R1 | 5’ ACTGACCTTTGAGCACAGAC 3’ |
| β-fibrinogen | Intron 7 | 614 | BFIB-R1 | 5’ ATTCACAACGGCATGTTCTTCAG 3’ |
|  |  |  | BFIB-R2 | 5' AANGKCCACCCCAGTAGTATCTG 3’ |
| Vimentin | Intron 8 | 785 | VIM-E8 F1 | 5’ AGAACACTCCTGATTAAGACG 3’ |
|  |  |  | VIM-E9 R1 | 5’ GCATCACGATGACCTTGAATAA 3’ |
| LCAT | Exons 3-6, introns 3-5 | 456 | LCAT-F2 | 5’ CTGGTACAGAATCTGGTTAAC 3’ |
|  |  |  | LCAT-R5 | 5’ TAAGACATCCTAATGGTGCTG 3’ |
| RAG1 | Exon 1 | 1183 | S77 | 5’ TCCATGCTTCCCTACTGACCTG 3’ |
|  |  |  | S71 | 5’ TGGCTTCTGGTTATGGAGTGGA 3’ |
| AP5 | Exons 2-3, intron 2 | 385 | AP5120-F1 | 5’ AATGCCCCATTCCACACAGC 3’ |
|  |  |  | AP5 564-R1 | 5’ GCAGAGACGTTGCCAAGGTG 3’ |
